# Positive Emotional Responses to Socially Assistive Robots in People With Dementia: Pilot Study

**DOI:** 10.2196/52443

**Published:** 2024-04-11

**Authors:** Eri Otaka, Aiko Osawa, Kenji Kato, Yota Obayashi, Shintaro Uehara, Masaki Kamiya, Katsuhiro Mizuno, Shusei Hashide, Izumi Kondo

**Affiliations:** 1 Laboratory of Practical Technology in Community, Assistive Robot Center, National Center for Geriatrics and Gerontology Research Institute, Obu, Aichi, Japan; 2 Department of Rehabilitation Medicine, National Center for Geriatrics and Gerontology, Obu, Aichi, Japan; 3 Laboratory of Cognitive Rehabilitation and Robotics, Assistive Robot Center, National Center for Geriatrics and Gerontology Research Institute, Obu, Aichi, Japan; 4 Laboratory of Clinical Evaluation with Robotics, Assistive Robot Center, National Center for Geriatrics and Gerontology Research Institute, Obu, Aichi, Japan; 5 Department of Rehabilitation, Fujita Health University Hospital, Toyoake, Aichi, Japan; 6 Faculty of Rehabilitation, Fujita Health University School of Health Sciences, Toyoake, Aichi, Japan; 7 Department of Physical Rehabilitation, National Center Hospital, National Center of Neurology and Psychiatry, Kodaira, Tokyo, Japan; 8 Department of Rehabilitation Medicine, Tokai University School of Medicine, Isehara, Kanagawa, Japan; 9 Assistive Robot Center, National Center for Geriatrics and Gerontology Research Institute, Obu, Aichi, Japan

**Keywords:** dementia care, robotics, emotion, facial expression, expression intensity, long-term care, sensory modality, gerontology, gerontechnology

## Abstract

**Background:**

Interventions and care that can evoke positive emotions and reduce apathy or agitation are important for people with dementia. In recent years, socially assistive robots used for better dementia care have been found to be feasible. However, the immediate responses of people with dementia when they are given multiple sensory modalities from socially assistive robots have not yet been sufficiently elucidated.

**Objective:**

This study aimed to quantitatively examine the immediate emotional responses of people with dementia to stimuli presented by socially assistive robots using facial expression analysis in order to determine whether they elicited positive emotions.

**Methods:**

This pilot study adopted a single-arm interventional design. Socially assistive robots were presented to nursing home residents in a three-step procedure: (1) the robot was placed in front of participants (visual stimulus), (2) the robot was manipulated to produce sound (visual and auditory stimuli), and (3) participants held the robot in their hands (visual, auditory, and tactile stimuli). Expression intensity values for “happy,” “sad,” “angry,” “surprised,” “scared,” and “disgusted” were calculated continuously using facial expression analysis with FaceReader. Additionally, self-reported feelings were assessed using a 5-point Likert scale. In addition to the comparison between the subjective and objective emotional assessments, expression intensity values were compared across the aforementioned 3 stimuli patterns within each session. Finally, the expression intensity value for “happy” was compared between the different types of robots.

**Results:**

A total of 29 participants (mean age 88.7, SD 6.2 years; n=27 female; Japanese version of Mini-Mental State Examination mean score 18.2, SD 5.1) were recruited. The expression intensity value for “happy” was the largest in both the subjective and objective assessments and increased significantly when all sensory modalities (visual, auditory, and tactile) were presented (median expression intensity 0.21, IQR 0.09-0.35) compared to the other 2 patterns (visual alone: median expression intensity 0.10, IQR 0.03-0.22*; P*<.001; visual and auditory: median expression intensity 0.10, IQR 0.04-0.23; *P*<.001). The comparison of different types of robots revealed a significant increase when all stimuli were presented by doll-type and animal-type robots, but not humanoid-type robots.

**Conclusions:**

By quantifying the emotional responses of people with dementia, this study highlighted that socially assistive robots may be more effective in eliciting positive emotions when multiple sensory stimuli, including tactile stimuli, are involved. More studies, including randomized controlled trials, are required to further explore the effectiveness of using socially assistive robots in dementia care.

## Introduction

The number of people with dementia is increasing globally, and it is estimated that it will reach 152 million by 2050 [1]. The provision of adequate social care for people with dementia is a major public health concern in many countries. The neurodegenerative nature of dementia affects memory, cognitive function, and more, resulting in a range of noncognitive symptoms, including changes in behavior, emotion, and social functioning. The most frequent changes include agitation, depression, and apathy. These emotional changes are reported as the most challenging aspect of dementia care by many caregivers [2]. On the other hand, positive emotions such as joy or comfort are relatively preserved until the terminal stage of the disease [3,4]. Previous studies suggest that the arousal of positive emotions may enhance cognitive function, presumably through amygdala activation; therefore, stimuli with a positive valence may enhance the effect of the rehabilitative approach for patients with dementia [3,5]. Considering these facts, interventions and caregiver involvement that can evoke positive emotions and suppress negative psychological responses are important strategies that should be actively implemented in long-term care for people with dementia to maintain residual functions and alleviate the burden of care.

As examples of emotion-related interventions, music therapy [6,7] and occupational therapy [8,9] have been shown to be effective in terms of emotional control. However, due to the rapid increase in the number of people with dementia and the shortage of dementia caregivers [1,10,11], there is a lack of staffing power to provide such nondrug therapies broadly and equally. In recent years, clinical applications of socially assistive robots have been used to provide high-quality emotional support and companionship [12-14]. Socially assistive robots are machines designed to provide assistance in the caregiving process through social rather than physical means and are equipped with a social interface to enable interaction with the user [15-17]. One systematic review and meta-analysis, as well as one scoping review, found that Paro, a baby seal–shaped socially assistive robot, has significant effects on agitation and depression [12,18], while another systematic review and meta-analysis concluded that there is little evidence that people with dementia derive benefits from socially assistive robots for cognition or neuropsychiatric symptoms when considering various types of robots, although they are feasible and acceptable [11]. On the other hand, one small between-groups comparison study reported that a certain type of socially assistive robot showed a negative effect in participants with cognitive decline, based on an examination of immediate neurophysiological changes [19].

When using socially assistive robots in clinical practice, one important aspect to consider is the immediate response of persons with dementia. In general, when confronted with a new robot or technology, a relatively positive immediate response known as the novelty effect [20] tends to be observed. In contrast, in people with cognitive decline, the immediate response to robots is reported to be somewhat stressful rather than positive [19]. These findings suggest that people with dementia, or those with memory and other cognitive impairments, may have a different immediate response compared to the general public. For example, people with dementia have a reduced ability to process multiple sensory stimuli [21]; therefore, they might have difficulty accepting and integrating multiple unfamiliar stimuli (eg, shapes, lights, sounds, and touch) provided simultaneously by the robot in the first interaction. Since they are prone to mental stress when they do not understand a situation [22], these stimuli from the robots could cause a tense or negative response. Nonetheless, no studies have verified how socially assistive robots are perceived by people with dementia from the perspective of having to process multiple sensory modalities. Moreover, an immediate response from the person with dementia is crucial in clinical settings because it helps care providers confirm the effectiveness of the robot on the spot and make precise decisions about whether to continue using the robot. Therefore, it is beneficial for clinical applications to focus on understanding the immediate responses of people with dementia when they are given multiple sensory modalities from socially assistive robots.

For an objective and better understanding of these issues, the signs of emotional responses should be quantified using appropriate techniques. Given that verbal skills tend to be impaired in people with dementia [23,24], it is important to use not only self-reported outcomes but also objective measures that can be obtained with minimal burden. For example, in the field of psychology, facial expression is considered a differentiated indicator of inner emotions [25,26]. According to recent reports, analysis of facial imaging using facial expression analysis software is able to quantify facial expressions and estimate emotions with good validity [27,28]. Applying these technologies to the investigation of the use of socially assistive robots among people with dementia will enable detailed and empirical verification of their effects, such as responses to the different sensory stimuli mentioned above, which are difficult to detect with subjective scales.

In this context, this study aimed to quantitatively evaluate the psychological and emotional reactions evoked in people with dementia to stimuli derived from socially assistive robots using facial expression analysis of facial video clips. In particular, we investigated how immediate responses changed as the modalities of sensory stimulation provided by the robot increased. Furthermore, from the perspective of eliciting positive emotions, which are beneficial to dementia care, this study also examined the differences in the emotion of joy elicited by the different types of robots.

## Methods

### Ethical Considerations

The study protocol was approved by the institutional ethics committee of the National Center for Geriatrics and Gerontology (1539) and prospectively registered in the UMIN Clinical Trial Registry (UMIN000046256). All participants with a Clinical Dementia Rating (CDR) scale [29] score of 0 or 1 provided informed consent themselves in accordance with the Declaration of Helsinki. For those who were considered to have an insufficient capacity to consent due to cognitive decline equivalent to CDR 2 and 3, informed consent was obtained from their family members, and the procedures were explained to the participants in plain language to obtain their approval. To comply with ethical principles, all data collected were anonymized and stored in a locked file or on a password-protected computer.

### Study Design and Setting

This pilot study was conducted as a single-arm, self-controlled, interventional study. Two local nursing care facilities that had no previous experience implementing socially assistive robots were selected as the experiment sites.

### Participants

Participants were recruited among the residents of the 2 nursing homes. The inclusion criteria were as follows: a significant decline in cognitive function interfering with independence in the performance of everyday activities, the ability to maintain a sitting position for 15 minutes or more, the ability to communicate using simple words, and the ability to follow 2-step instructions. These criteria were first assessed by nursing home staff members through assessments performed as part of daily nursing care procedures. Regarding cognitive decline, candidates either had a previous formal diagnosis of dementia from their physician or received a diagnosis from one of the researchers (EO, a physician); they also had confirmed evidence that cognitive decline was present and that the decline was not due to delirium or other mental disorders. Ultimately, all the participants met the diagnostic criteria for dementia in the *Diagnostic and Statistical Manual of Mental Disorders, Fifth Edition* (*DSM-5*). Those with unstable physical or mental conditions or evident higher cognitive dysfunction due to causes other than dementia were excluded.

### Procedures

**Figure 1.**
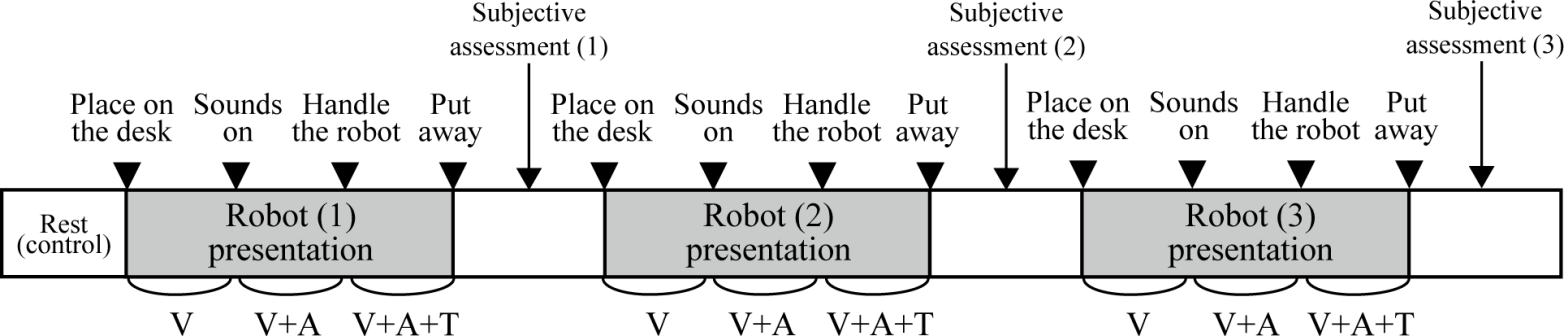
The flow of robot presentation to the participant. V: visual stimulus only; V+A: visual and auditory stimuli; V+A+T: visual, auditory, and tactile stimuli.

The participants were taken to a private room or a place with minimum environmental noise, and their faces were recorded in a resting state for 30 seconds. This was referenced as the control image for calibrating the facial expression analysis (to be described below). Next, the socially assistive robots were presented to the participant by a familiar staff member in 1 session using a predetermined 3-step procedure. The staff explained in advance that they wanted the participants to share how they felt after experiencing the robots. In the first step, the robot was placed on a desk in front of the participant (visual stimulus). In the second step, the robot was manipulated to produce a gentle voice or meow (visual and auditory stimuli). In the third step, the participant was encouraged to touch the robot (visual, auditory, and tactile stimuli) and was able to handle it freely, including petting and holding. The 3 patterns of sensory stimulus produced by the robots were presented for approximately 30 seconds in the context of assessing the participant’s immediate responses unless the participant refused (Figure 1).

**Figure 2.**
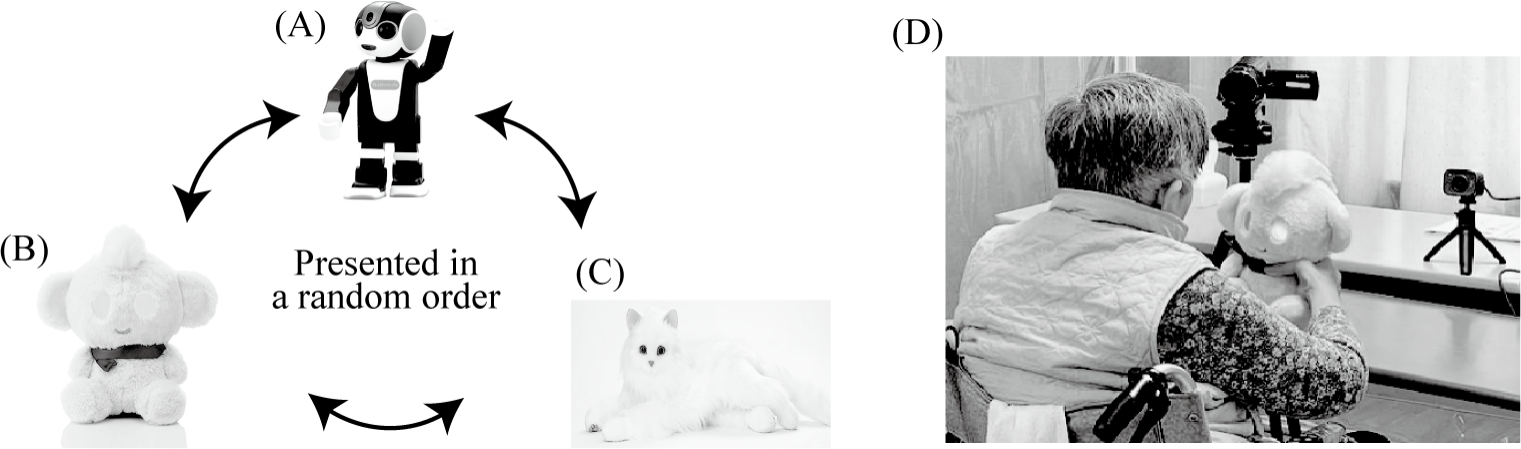
Socially assistive robots used in this study. Each participant was presented with these 3 types of robots with typical characteristics, resulting in 3 sessions per participant. The order of presentation was determined in advance using a random number table. (**A**) Humanoid-type, (**B**) doll-type, and (**C**) animal-type robots; (**D**) video recording while the participant is touching the robot.

Each participant was presented with 3 types of commercially available robot with typical characteristics—a humanoid-type robot capable of voice communication (RoBoHoN; Sharp Corp), a doll-type robot with the appearance of a stuffed toy and a voice recognition and reproduction system (Chapit; RayTron Inc), and an animal-type (cat-shaped) robot that can meow, move its tail, and recognize sound (Amaenbou-Nekochan; Digirect Co, Ltd)—resulting in 3 sessions per participant. The order of presentation, which was determined in advance using a random number table, differed for each participant (Figure 2).

### Assessments of Emotional Responses

#### Subjective Assessment

At the end of each robot presentation session, the degree of subjective feelings (happy, sad, angry, surprised, scared, and disgusted) was recorded using a 5-point Likert scale (1: not at all; 2: very little; 3: so-so; 4: somewhat; 5: very much). The participants were shown a scale with words corresponding to each of the 5 points of the scale and were asked to select the point that best described their current feelings. This was assessed a total of 3 times (ie, once at the end of each robot presentation).

#### Video Recording and Facial Expression Analysis

For the study procedures, a video camera (Logicool StreamCam; Logitech Inc) was configured on a desk to capture the participants’ faces from the front. In case this camera’s view was blocked by arm movements or the robot, another camera (Handycam HDR-CX470, Sony Corp) was fixed on a tripod positioned 30 degrees diagonally in front of the participant.

The videos were recorded continuously during the session in full HD (1920 × 1080 pixels) at 60 frames per second. To optimize the sensitivity and accuracy of the facial expression analysis, we segmented the video clips such that each clip contained 1 sensory stimulation pattern (visual only; visual and auditory; or visual, auditory, and tactile), and then cropped them to a suitable resolution that focused on the facial region. If necessary, the brightness of the video clips was minimally adjusted.

We used commercially available software for quantifying facial expressions (FaceReader; version 7; Noldus Information Technology Inc). This software was developed based on a quantitative evaluation method called the Facial Action Coding System [30], which describes visually identifiable facial muscle movements as “action units,” identifies the intensity of a basic emotional state, and outputs time-series data sets comprising expression intensity values from 0 to 1 for each of the 7 facial expression elements (ie, neutral, happy, sad, angry, surprised, scared, and disgusted) on a continuous scale with high accuracy [27,31,32]. This method is advantageous in that it can quantitatively evaluate facial expressions conveniently with good reproducibility, unlike subjective evaluations, as discussed previously [33]. The software provides 5 face models (General, General61, Children, East Asians, and Elderly) that correspond to the data sets used in the algorithm training. We used the East Asian face model according to the software specifications.

Additionally, we used the calibration function provided by the software to minimize person-specific biases due to facial wrinkles or light effects. The reference manual of FaceReader explains that this function removes biases in the 7 facial expression elements but does not increase the intensity. For each participant, the resting facial image in the first part of recording was used as the calibration image for all the video clips of the participant. In cases where certain facial expression elements were detected in the neutral control image, those expression elements were corrected in the images to be analyzed. The degree of successful face recognition was evaluated for every video clip, and the session was excluded from further analysis if both images from the 2 cameras had a low proportion of successful frames (<20%), with reference to previous studies [32,34].

#### Clinical Assessments

In addition to basic characteristics, overall cognitive function was assessed using the Japanese version of the Mini-Mental State Examination (MMSE-J) [35] and the Japanese version of the Montreal Cognitive Assessment (MoCA-J) [36]. The Barthel index was used to assess performance on 10 basic activities of daily living (ADL), which tend to deteriorate in people with dementia. The total score ranges from 0 (worst; all dependent) to 100 (best; all independent). The Dementia Behavior Disturbance Scale (DBDS) [37,38] was used to assess the severity of neuropsychiatric symptoms. The scale evaluates a total of 28 items on a 5-point scale from 0 (not at all) to 4 (always) in terms of the frequency of the behavioral disturbances typically seen in persons with dementia, such as wandering, agitation, and aggression, and is scored from 0 (best) to 112 (worst). The questionnaire format allowed caregivers to answer the questions easily. Finally, to assess hearing disability, which may affect the response to auditory stimulation, 10 items from the Questionnaire on Hearing [39] were surveyed to score the severity of hearing loss in daily life. These 10 questions set up specific situations of hearing speech or environmental sounds in daily life and were to be answered on a 5-point scale from 1 (always able to hear) to 5 (never able to hear). The total score ranges from 10 (best) to 50 (worst).

The MMSE-J and MoCA-J were administered by skilled occupational therapists on different days. The Barthel index, DBDS, and the Questionnaire on Hearing were scored by nursing home staff members who were sufficiently familiar with the participants.

### Statistical Analyses

The averages of the emotions expressed in response to the robot (ie, happy, sad, angry, surprised, scared, and disgusted) were compared to each other using the nonparametric Wilcoxon signed-rank test, with the *P* values multiplied by the number of tests according to the Bonferroni method. The correlations between subjective and objective emotional assessments were also examined using Spearman correlation coefficients. The average expression intensity values during the 30 seconds for each sensory stimulus obtained by facial expression analysis were compared for all 3 patterns (visual only; visual and auditory; or visual, auditory, and tactile) using the Friedman test with the Wilcoxon signed-rank test as a post hoc test. The average of the 3 robot presentation sessions was used in this part of the analysis. Additionally, focusing on positive emotions, the expression intensity values for “happy” were compared between the different types of robots using the Friedman test with the Wilcoxon signed-rank test as a post hoc test. Statistical analyses were performed using STATA/SE (version 13.1; StataCorp). Any *P* value less than .05 was considered statistically significant.

## Results

Table 1 presents the demographic characteristics of the participants. Eleven participants (38%) had been diagnosed with Alzheimer disease by their physician, 5 (17%) had dementia with Lewy bodies, and 13 (45%) met the criteria for major neurocognitive disorder in the *DSM-5*, but the etiology was not specified. All the participants had cognitive decline when compared with the cutoff value of 26 on the MoCA-J [36].

**Table 1.** Overall participant characteristics (N=29).

Characteristics		Values
Age (years), mean (SD; range)		88.7 (6.2; 71-98)
**Gender, n**		
	Male	2
	Female	27
**Type of disease, n**		
	Alzheimer disease^a^	11
	Dementia with Lewy bodies^b^	5
	Not specified	13
Years of education, mean (SD; range)		9.7 (2.2; 6-13)
MMSE-J^c^, mean score (SD; range)		18.2 (5.1; 11-28)
MoCA-J^d^, mean score (SD; range)		11.8 (4.9; 2-24)
Barthel index, mean score (SD; range)		66.0 (24.8; 10-95)
10 items from the Questionnaire on Hearing, mean score (SD; range)		26.6 (8.6; 14-50)
DBDS^e^, mean score (SD; range)		13.4 (9.6; 0-40)

a A total of 4 patients were taking medication for dementia.

b A total of 2 patients were taking medication for dementia.

c MMSE-J: Japanese version of Mini-Mental State Examination.

d MoCA-J: Japanese version of Montreal Cognitive Assessment.

e DBDS: Dementia Behavior Disturbance Scale.

**Figure 3.**
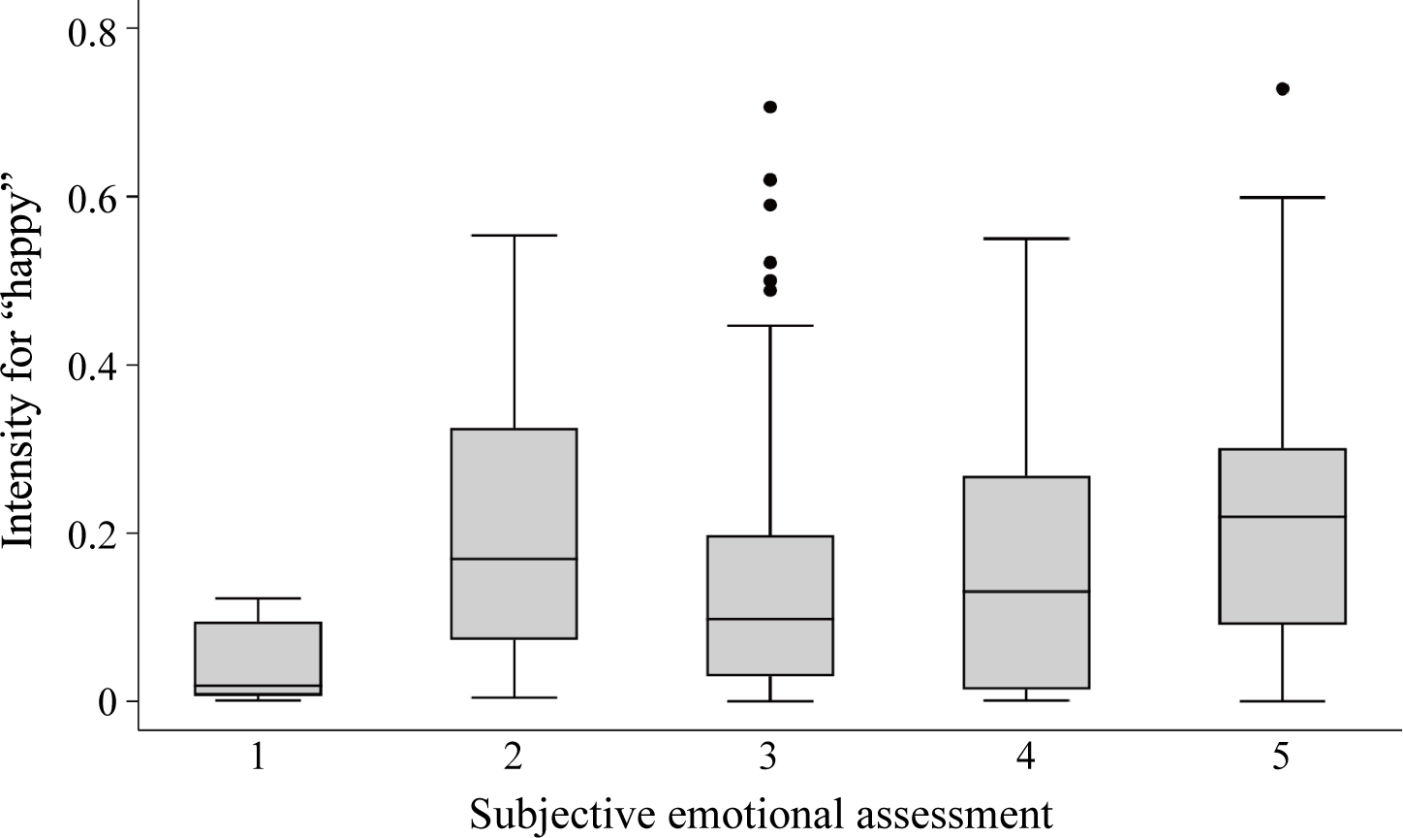
Comparison of the objective expression intensity for “happy” at each grade of subjective emotional assessment (5-point Likert scale). Error bars indicate SDs, the lines within the boxes indicate medians, and the dots indicate outliers that were more than a quarter of the range × 1.5 away from the first or third quartile.

The average subjective emotional assessments (5-point Likert scale) and objective expression intensity values for the expression elements across all of 3 types of robots are shown in Table 2. Facial analysis failed to detect any action units or emotional elements in 1 of the 29 participants. Also, 1 video clip was excluded from the analyses because of a low proportion of successful frames (participant 13; doll-type robot; visual, auditory, and tactile stimuli presented). After excluding these video clips, the overall percentage of the video frames analyzable by the software was 81.4%. Among the self-reported emotions, “happy” was significantly the most common (happy vs surprised: *P*=.01; happy vs sad, angry, scared and disgusted: *P*<.001), and facial analysis–detected emotions showed the same trend in that the values of “happy” were significantly the most common among the 6 emotional elements assessed (happy vs all others: *P*<.001). Additionally, the correlations between subjective and objective emotional assessments were significant for “happy,” “sad,” and “surprised,” though the correlation coefficients were interpreted as slight or low. Taking the value of “happy” as an example, as shown in Figure 3, there were cases where the objective value was detected as high even when the subjective feelings were reported as low.

**Table 2.** The relationship between subjective and objective emotional assessments.

	Subjective (5-point Likert scale; n=29)		Objective (expression intensity values; n=28)		ρ	*P* value
	Mean (SD)	Range	Mean (SD)	Range		
Happy	3.6 (1.0)	1-5	0.18 (0.16)	0-0.73	0.21	<.001
Sad	2.1 (0.8)	1-5	0.07 (0.09)	0-0.61	0.14	.03
Angry	1.9 (0.8)	1-4	0.07 (0.11)	0-0.77	0.06	.31
Surprised	3.1 (1.1)	1-5	0.09 (0.11)	0-0.62	0.29	<.001
Scared	1.9 (0.7)	1-5	0.03 (0.05)	0-0.36	0.05	.42
Disgusted	2.0 (1.0)	1-5	0.05 (0.06)	0-0.34	0.08	.21
Neutral	N/A^a^	N/A	0.46 (0.13)	0.14-0.85	N/A	N/A

a N/A: not applicable.

**Figure 4.**
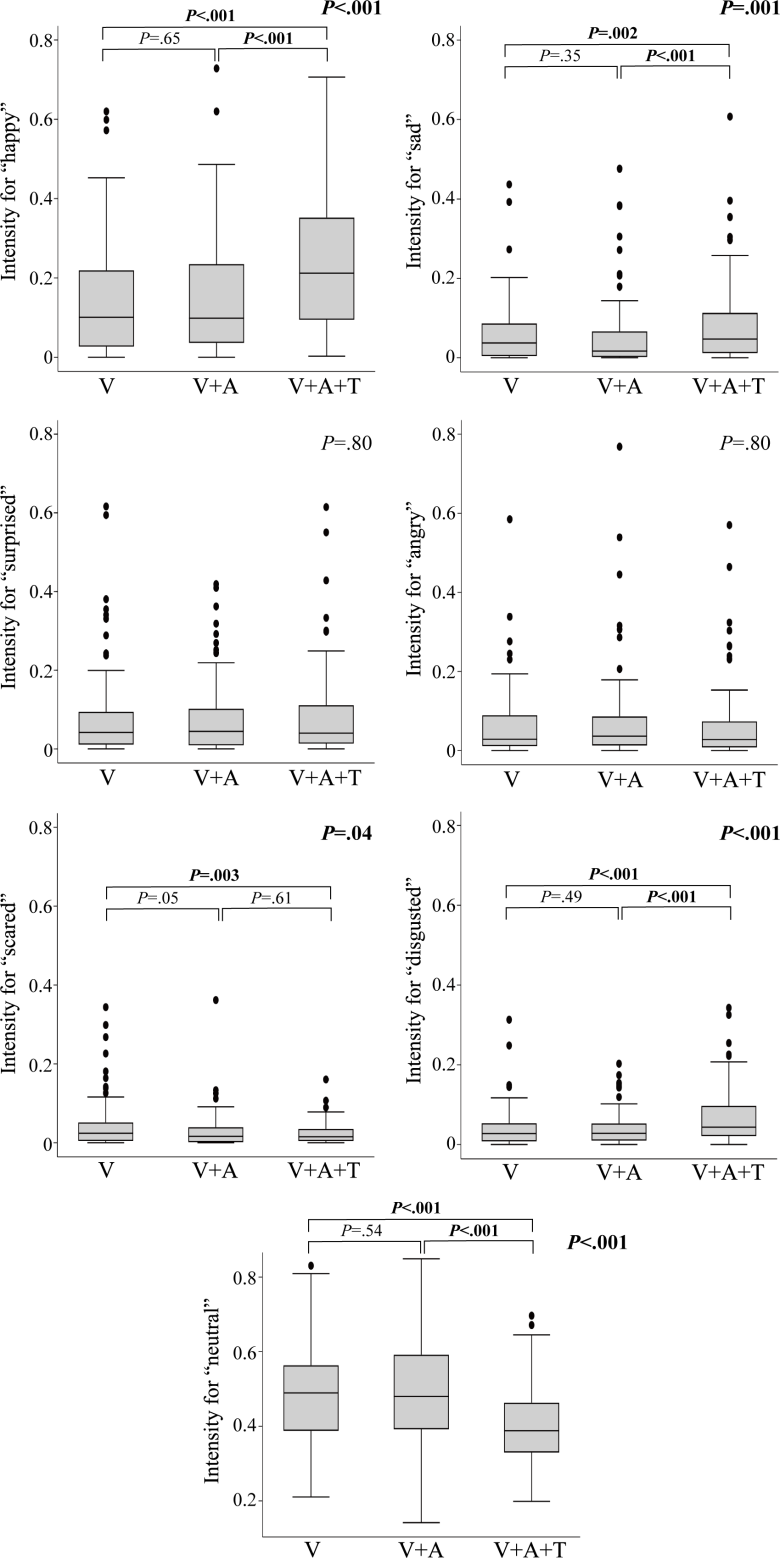
Comparison of all the expression intensities under the 3 different patterns of sensory stimulation. Error bars indicate SDs, the lines within the boxes indicate medians, and the dots indicate outliers that are more than a quarter range × 1.5 away from the first or third quartile. The values in the upper right of each group represent the overall *P* values in the Friedman test. The values above the boxplots for the nonresponder group represent the *P* values in the post hoc test (the Wilcoxon signed-rank test). *P* values less than .05 are denoted in bold. V: visual stimulus only; V+A: visual and auditory stimuli; V+A+T: visual, auditory, and tactile stimuli.

Figure 4 shows a comparison of 3 different patterns of sensory stimuli among all participants. There were significant differences in the values for “neutral” (*P*<.001), “happy” (*P*<.001), “sad” (*P*=.001), “scared” (*P*=.04), and “disgusted” (*P*<.001) among the 3 patterns. Post hoc analyses revealed that the values for “happy” significantly increased in the pattern with visual, auditory, and tactile stimuli (median score 0.21, IQR 0.09-0.35) compared to the patterns with visual stimulus only (median score 0.10, IQR 0.03-0.22; *P*<.001) and with both visual and auditory stimuli (median score 0.10, IQR 0.04-0.23; *P*<.001). The values for “sad” (with visual, auditory, and tactile stimuli: median score 0.05, IQR 0.01-0.11) and “disgusted” (with visual, auditory, and tactile stimuli: median score 0.04, IQR 0.02-0.10) exhibited the same trend, though both of these values were significantly smaller than those for “happy” (*P*<.001). In contrast, the values for “neutral” and “scared” significantly decreased in the pattern with visual, auditory, and tactile stimuli compared with the other 2 patterns. However, the intensity of each emotional element did not change linearly over time.

**Figure 5.**
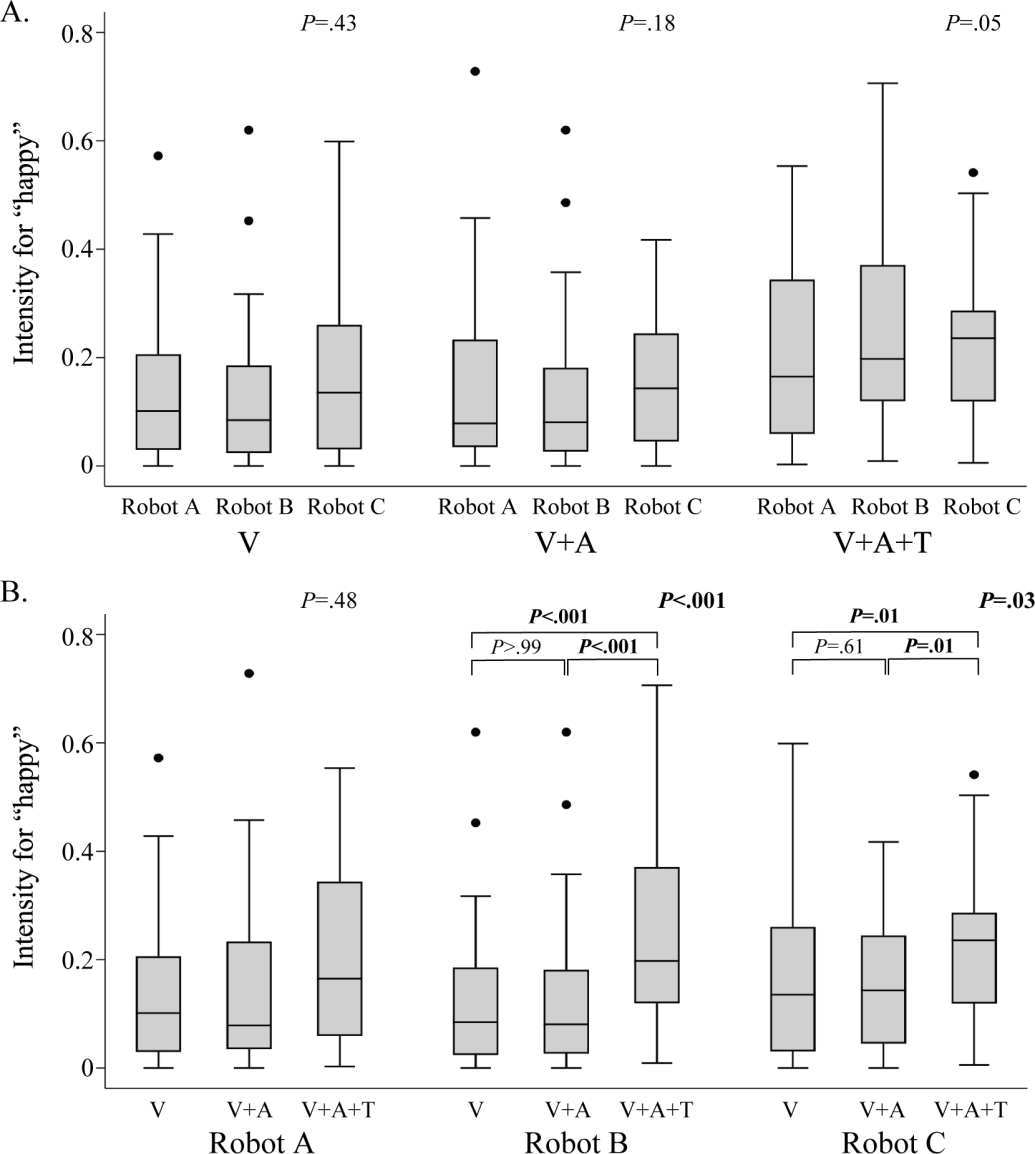
Comparison of the expression intensity for “happy” between the different types of robots. (A) Comparison between 3 types of robots with the same pattern of sensory stimulation. (B) Comparison within each robot type using the 3 different patterns of sensory stimulation. Error bars indicate SDs, the lines within the boxes indicate medians, and the dots indicate outliers that are more than a quarter of the range × 1.5 away from the first or third quartile. The values in the upper right of each group represent the overall *P* values from the Friedman test. The values above the boxplots for robots B and C represent the *P* values from the post hoc test (the Wilcoxon signed-rank test). *P* values less than .05 are denoted in bold.

When comparing the expression intensity values for “happy” between the different types of robots for the same participants and the same sensory stimuli, no statistical differences were found, as depicted in Figure 5A. When comparing the expression intensity values for “happy” between the different sensory stimuli for the same robot type, no statistically significant differences were found for robot A (humanoid-type*; P*=.48), while robot B (doll-type*; P*<.001) and robot C (animal-type*; P*=.03) had large, significant values in the pattern with visual, auditory, and tactile stimuli compared with the other 2 patterns, as shown in Figure 5B.

## Discussion

### Principal Results

This study aimed to quantitatively evaluate the psychological and emotional responses of people with dementia to complex sensory stimuli provided by socially assistive robots. To summarize, the values for “happy” were found to be the largest in the overall response to the robots among the emotional elements in both the self-recorded assessment and objective facial expression analysis. However, correlations between the subjective and objective emotional assessments were found to be relatively low or insignificant. When comparing the 3 different patterns of sensory stimulation, there was a significant increase in some of the expression intensity values when all stimuli (visual, auditory, and tactile) were presented. In the comparison between different types of robots, focusing on “happy,” there was a significant increase when all stimuli were presented by a doll-type robot and by an animal-type robot but not by a humanoid-type robot.

The emotional responses revealed by the objective facial expression analysis showed the same trend as the participants’ subjective assessment, with happy emotions having the largest values in both assessments. This result suggests that the facial expression analysis system can successfully detect the expressions that emerge in people with dementia, which is consistent with a previous study [40].

However, considering that the facial expression intensity was detected as relatively high in some cases where the subjective rating was moderate, the facial expression analysis may be able to capture emotions that are not sufficiently represented by a subjective 5-point Likert scale. In fact, a previous study supports the appropriateness of facial expression analysis as a nonverbal pain assessment for people with dementia when they lack the ability to self-report [41]. Therefore, facial expression analysis may also be useful as an accurate nonverbal assessment of emotions for people with dementia.

Although some studies have already demonstrated positive responses in people with dementia when presented with socially assistive robots [42,43], this study is the first to objectively and quantitatively describe emotional responses using facial expression analysis. Additionally, these results are inconsistent with those of Goda et al [19], who found that a 5-minute talk session with a socially assistive robot caused stress in people with dementia. In contrast to this study, the socially assistive robot’s interaction in the study by Goda et al [19] was mainly through verbal communication; thus, it was inferred that these stimuli were burdensome for people with dementia, who tend to have a decline in verbal communication skills. The positive expressions shown in this study are considered to have been observed as responses to sensory stimuli and not verbal stimuli.

Regarding the relationship between the type of sensory stimulus and emotional responses, the expression intensity values for “happy,” “sad,” and “disgusted” significantly increased with increasing sensory stimuli. Particularly, happy emotions were revealed to be elicited most strongly in persons with dementia when different varieties of sensory stimuli were presented. With respect to the values for “sad” and “disgusted,” we consider it natural that participants became somewhat careful or timid toward unknown experiences when interacting closely with robots. However, these emotional elements in automated analysis should be interpreted with caution, as they can occasionally include other negative emotions, such as fear [44], especially when accompanied by facial movements around the lips or jaws [31]. Nevertheless, the values for these negative emotions remain small compared to the value for “happy,” showing that positive emotions were dominant when visual, auditory, and tactile sensory stimuli were offered.

Notably, this increase in positive emotional responses may include changes over time, because the sensory stimuli were added over time in this study. Given the procedures in this study, the results could have been influenced by the novelty effect [20], which is derived from curiosity toward a new experience. However, the intensity of each emotional element did not change linearly when sensory stimuli were added over time. Moreover, the addition of an auditory stimulus did not significantly increase the expression intensity value, whereas the addition of a tactile stimulus did. These findings suggest the importance of adding tactile stimuli to visual and auditory stimuli. This is plausible considering that tactile information connects through several pathways to the insular cortex, which evokes emotional responses [45,46]. The finding that tactile stimuli evoked positive feelings is supported by previous studies that showed that haptic or tangible input was effective in helping people with dementia understand and adapt to their surroundings [47,48]. Regarding auditory stimuli, the results of the Questionnaire on Hearing did not indicate that the participants had very good hearing, suggesting that the effects of auditory stimuli on emotion elicitation may have been relatively small. Since hearing loss is associated with the risk of developing dementia [49], and a high percentage of people with dementia actually have hearing loss [50,51], this issue may also be of great clinical importance.

Furthermore, it is noteworthy that a significant increase in expression intensity values for “happy” with increased sensory stimuli was observed for doll-type robots and animal-type robots, but not for humanoid-type robots. One of the distinctive characteristics of the doll-type and animal robots used in this study was that they were covered by soft, fur-like materials. The importance of soft materials is commonly discussed in the field of soft robotics for medical use or human assistance [52,53]. Softness is considered effective not only in terms of safety for the human body but also in terms of the imitation of reality or the creation of familiarity [54] and emotional processing [55]. However, the emotional effects of various tactile sensations in people with dementia have not yet been studied; consequently, given the findings of this study, the effectiveness of soft tactile stimuli in dementia care may be worth exploring in future research.

### Limitations

This study has a few limitations. First, it included a small number of participants and a single experimental group. Changes in facial expressions were reliably detected by using the participants’ resting states as controls. However, another study design, such as a randomized controlled trial, is required to confirm these effects more clearly. Second, the generalizability of our findings may be limited, as most of the participants in this study were female. Previous studies report that there are gender differences in emotional responses to some types of sounds [56], emotion expression [57], and emotion regulation [58], although gender and facial expression have been reported to have no significant correlation [40]. Further research with male participants will be needed to reveal possible gender differences in responses toward social robots. Third, since there is no prior literature that has identified a minimum detectable change or a minimal clinically important difference for expression intensity, the clinical significance of the changes in expression intensity values demonstrated in this study needs to be explored further. Finally, this study only investigated the immediate responses to socially assistive robots, with patients allowed to interact with each robot for only 1.5 minutes in total. However, in real clinical settings, people with dementia might express more diverse patterns of responses, using these robots as they would like. Moreover, any enthusiasm resulting from the novelty effect may diminish over time. Thus, further investigation is required to reveal the long-term emotional effects of socially assistive robots on people with dementia, including variability in positive responses over several hours or days of use and the effects of these robots on their neuropsychiatric symptoms.

### Conclusions

This study quantitatively examined the emotional reactions of people with dementia to socially assistive robots. The expression intensity values, especially the values for “happy,” significantly increased with multiple sensory stimuli, including visual, auditory, and tactile stimuli. Therefore, this study shows that socially assistive robots may be more effective in arousing positive emotions when multiple sensory stimuli are involved. Further studies, including randomized controlled trials, are required to further explore the effectiveness of and the optimal methods for using socially assistive robots in dementia care.

## Abbreviations

ADL: activities of daily living
CDR: Clinical Dementia Rating
DBDS: Dementia Behavior Disturbance Scale
*DSM-5*: *Diagnostic and Statistical Manual of Mental Disorders, Fifth Edition*
MMSE-J: Japanese version of Mini-Mental State Examination
MoCA-J: Japanese version of Montreal Cognitive Assessment
